# Shaping a healthier digital food environment in Malaysia: a call for nutrition-conscious action

**DOI:** 10.1017/S1368980026102626

**Published:** 2026-05-15

**Authors:** Seok Tyug Tan

**Affiliations:** Jeffrey Cheah School of Medicine and Health Sciences, Monash University Malaysia, Malaysia

**Keywords:** Digital food environments, Nutrition-conscious action, Dietary recommendations, Malaysia

## Abstract

Although the shift from traditional to digital food environments has enhanced accessibility and convenience in food purchasing and delivery, emerging evidence suggests that current digital food environments tend to promote energy-dense foods rather than healthier options. Therefore, this commentary aims to highlight the challenges faced by healthcare professionals in shaping a healthier digital food environment and to offer practical recommendations for fostering such an environment in Malaysia. This commentary also emphasises that shaping a healthier digital food environment is not an individual endeavour but a collaborative effort among key stakeholders, including government ministries, platform operators, online food vendors and platform users.

According to the ICT Use and Access by Individuals and Households Survey Report (2024), over 90 % of Malaysians aged 15 years and above have internet access and own a mobile phone^(1)^. The widespread digital connectivity may have shaped food-related decisions, eating behaviours and even the nutritional status of Malaysians. For instance, Eu & Sameeha^(2)^ reported that slightly more than three-quarters of Malaysian university students ordered energy-dense foods through online food delivery applications due to the limited availability of healthy options on these platforms. Such findings underscore the urgent need to foster a healthier digital food environment for younger generations in Malaysia.

In this context, the digital food environment refers to online platforms through which flows of information and services that may influence individuals’ food choices, eating behaviours and nutritional outcomes are directed^(3)^. Based on the framework suggested by Granheim *et al.* ^(4)^, it encompasses digital food availability, food retail and delivery, food choice and purchasing, food and health information, social engagement, food entertainment, meal planning and dietary tracking, and food marketing. Although the shift from traditional to digital platforms has increased accessibility and convenience in purchasing and delivery^(5)^, it also presents substantial challenges for healthcare professionals in shaping a healthier digital food environment. Therefore, this commentary aims to highlight these challenges and offer recommendations for fostering such an environment in Malaysia.

## Challenges encountered by healthcare professionals in the digital food environment

In Malaysia, food items ranging from raw ingredients to ready-to-eat meals are increasingly sold, delivered and marketed through digital platforms. These platforms may include, but are not limited to, multi-vendor e-commerce marketplaces (such as Shopee, Lazada and Taobao), first-party online retailers (such as Lotus’s online and MyAeon2go), first-party food service providers (such as McDonald’s, KFC and Domino’s) and third-party online food delivery platforms (such as FoodPanda, GrabFood and ShopeeFood). Although social media platforms (such as Instagram, TikTok and YouTube) engage in food selling to a lesser extent than other digital platforms mentioned above, they primarily serve as key channels for food marketing and promotion. Thus, the challenges discussed in this commentary are centred on the operation and impact of these widely used digital platforms in Malaysia.

## Challenge 1: Food marketed on digital platforms may not meet safety, hygiene or labelling standards

In contrast to the traditional food environment, multi-vendor e-commerce marketplaces enable cross-border trade among local and international vendors. While this has increased food accessibility for Malaysians, it has also raised substantial concerns regarding food safety and hygiene due to the emergence of counterfeit food products. As counterfeit food products are unlikely to comply with Hazard Analysis and Critical Control Point or Good Manufacturing Practices standards, they may contain contaminants that cause adverse health effects.

Heavy reliance on social media platforms among Malaysians has also created opportunities for food handlers to sell homemade food online. This is typically facilitated by promoting and selling homemade food on social media platforms, with delivery arranged through third-party logistics providers such as Lalamove. Such a mode of operation poses several challenges related to food safety, hygiene and regulatory oversight. First, in addition to cooked food, third-party logistics providers are also permitted to transport perishable goods and non-food items. If cooked food is not separated from these items during transportation, cross-contamination may occur. Second, although third-party logistics providers offer a convenient way to trade homemade food, prolonged transportation times and the inappropriate selection of delivery vehicles by food handlers may compromise the safety and quality of time/temperature-controlled foods^(6)^. Third, while all food handlers are required to complete the Food Handler Training Course overseen by the Ministry of Health Malaysia (MOH) and to be vaccinated against typhoid before starting a food business, many home-based food handlers are unaware of these requirements^(7)^. As a result, their adherence to proper food hygiene standards remains questionable.

In Malaysia, imported foods sold on shelves must strictly comply with the Guidelines on Labelling Requirements under the Food Act 1983. For instance, the product name, ingredient list (including allergens), date marking, nutrition facts, manufacturer details and the name and address of the Malaysian importer must be presented in either *Bahasa Malaysia* (Malay) or English. In addition, any nutrition claims should adhere to the provided guidelines^(8)^. However, cross-border vendors may find it challenging to comply with these requirements due to the limited understanding of local food labelling requirements. Failure to comply with these labelling requirements not only poses potential food safety risks but may also result in misleading nutrition claims.

## Challenge 2: Exposure of children to unhealthy food promotions via digital marketing

As discussed earlier, social media platforms are the primary channels for food marketing and promotion. Unfortunately, emerging evidence suggests that such digital marketing extends beyond adult audiences to children. Unhealthy foods can be promoted in online videos through embedded content, video thumbnails or advertisements and by child influencers^(9)^. Children with limited cognitive ability may unknowingly adopt unhealthy dietary practices after watching such videos, thereby increasing their risk of childhood obesity^(10)^.

## Challenge 3: Commercial engagement strategies competing with health-oriented dietary decision-making

Digital food marketing strategies encompass direct brand–user interactions, gamification, point-collection systems, promotional vouchers and algorithm-driven personalised recommendations designed to stimulate food-purchasing intentions among digital platform users. These strategies are often implemented through influencer- and live-streamer-led marketing, engagement-based promotions (such as liking or tagging friends in food posts in exchange for rewards or price discount vouchers), gamified activities that allow users to earn points or vouchers, food-related entertainment content (including culinary livestreams and brand integration within online programmes) and user-generated content (such as reviews, ratings and food images or videos uploaded by the platform users)^(4)^.

Digital food marketing may negatively influence the food-related decisions of the platform users^(11)^. In Malaysia, a former study by Tan *et al.* ^(10)^ revealed that social media platforms are more likely to promote unhealthy foods and beverages than healthy options. Extensive digital food marketing may therefore prompt individuals to consume more unhealthy foods and beverages rather than make food-related decisions based on nutrition consciousness. Another concern worth highlighting is the involvement of influencers and live streamers in promoting dietary supplements on digital platforms. They often employ persuasive techniques such as personal testimonials, unsubstantiated claims and visual demonstrations to portray supplements as essential for health, fitness or wellness goals^(12)^. As these influencers and live streamers typically lack the nutrition knowledge to support their views, they may unintentionally disseminate exaggerated health claims not supported by scientific evidence.

## Challenge 4: Limited healthy options and lack of visible nutrition information

In Malaysia, Chin *et al.* ^(13)^ revealed that 65 % of common foods and 79 % of common beverages on third-party online food delivery platforms were deemed less healthy due to being energy-dense. Limited healthy options on these platforms may leave users with no choice but to order such foods for convenience. Moreover, the absence or incompleteness of nutrition labelling (such as front-of-pack labelling, nutrition information panels, and health claims) on digital platforms may hinder informed choices and further contribute to unhealthy dietary patterns among users.

## Challenge 5: Algorithmic recommendations deprioritise healthy choices

Recommendations in current online food delivery applications rely on machine learning-based recommender systems that suggest items based on users’ past order histories, ratings and reviews, as well as the real-time availability of food on the platform^(14)^. Consequently, items that are most frequently ordered, receive positive reviews and high ratings and are available at the time of ordering tend to appear at the top of recommendations, irrespective of their nutritional quality. Since energy-dense foods dominate ordering patterns on these platforms, these systems systematically prioritise such items while deprioritising nutritionally healthier options, such as fruits and vegetables^(15)^.

## Practical recommendations for shaping a healthier digital food environment

### Recommendation 1: Strengthening regulatory enforcement and compliance on digital platforms

Several initiatives can be implemented to prevent the proliferation of counterfeit products on online marketplaces. First, food manufacturers should strengthen surveillance and report unauthorised resellers of counterfeit products to the Food Safety and Quality Division (FSQD) under the MOH and the Ministry of Domestic Trade and Cost of Living. Second, food manufacturers can issue authenticity letters to authorised resellers and require them to display these letters on the product pages. Third, the FSQD may perform random product testing on items sold through digital platforms to verify the authenticity. Lastly, stricter law enforcement against counterfeit products must be implemented. Any unauthorised resellers found selling counterfeit products must be promptly prosecuted under relevant legislation (such as the Food Act 1983, the Food Regulations 1985, the Food Hygiene Regulations 2009 and the Consumer Protection Act 1999) to ensure accountability and consumer safety^(16)^.

To tackle issues related to non-compliance with the Food Handler Training Course and unvaccinated home-based food handlers, the MOH may collaborate with relevant digital platforms to monitor compliance and support the implementation of regulatory requirements. Existing home-based food handlers may be granted a 6-month compliance grace period to fulfil the requirements stipulated by the MOH. For new entrants intending to sell homemade food products, the relevant platforms must ensure that they have already complied with all regulatory requirements during the registration of their seller accounts. The MOH may also issue a certificate of compliance to home-based food handlers who have met all requirements. This certificate must then be submitted to the platforms to confirm their compliance. Home-based food handlers who fail to submit the required certificate should be prohibited from selling through digital platforms. At the same time, food delivery riders should be required to undergo the same Food Handler Training Course as food handlers to ensure that food ordered through digital platforms is maintained at appropriate temperatures during delivery.

### Recommendation 2: Parental and digital interventions to promote healthy eating in children

The Malaysian government has recently gazetted the Online Safety Act 2025 (ONSA 2025) to prohibit children under 16 years from registering or using social media accounts^(17)^. Although ONSA 2025 does not explicitly regulate digital food marketing, limiting social media access may reduce children’s exposure to unhealthy food marketing. As parents play a crucial role in shaping a child’s eating habits, nutritionists and dietitians can support and educate them to make healthier choices for their children. Parents can also supervise their children’s social media use and provide real-time guidance on unhealthy food content. Despite the widespread presence of unhealthy food content, social media could also offer an opportunity for nutritionists and dietitians to promote healthy options and assist children in recognising unhealthy foods. In this context, child-appropriate tools such as animations, gamification or child influencers can be employed to support these interventions. Moreover, school-based education programmes can be implemented by nutritionists or dietitians to support healthy decision-making in children.

### Recommendation 3: Incentivising healthier food choices on digital platforms

To mitigate the limited availability of healthier options and the widespread presence of energy-dense foods on digital platforms, the government may collaborate with online food vendors and marketplace operators to foster a healthier digital food environment. For example, free delivery vouchers, discount vouchers and gamification-based rewards are currently available for all food items on digital platforms, regardless of nutritional quality. The government could restrict such incentives to healthier options certified by nutritionists or dietitians. In addition, in-kind or financial incentives such as reducing platform commissions or providing marketing subsidies should be granted to vendors selling foods that align with healthy eating guidelines or nutrient-dense foods to increase the visibility of healthier choices on digital platforms.

### Recommendation 4: Enhancing labelling transparency and accountability on digital platforms

To address non-compliance with labelling requirements and misleading health claims on online marketplaces, the Malaysian government enacted the Consumer Protection (Electronic Trade Transaction) Regulations 2024 (CPETTR 2024)^(18)^. Under CPETTR 2024, all sellers and vendors must disclose their company details and present the product title and main description (including any health-related claims) in *Bahasa Malaysia* on the product page. Vendors and sellers of health supplements, over-the-counter medicines and food products are also required to provide a certificate confirming that these items comply with the safety and health standards set by the relevant competent authority. To further strengthen its public health impact, the government could augment CPETTR 2024 by mandating online vendors to display nutrition facts on product pages. The availability of such information can perhaps assist platform users in making informed choices. To reinforce compliance with local labelling and health claim standards among cross-border vendors, the government may provide online training to help them understand local regulations. Government-certified local agencies could also be assigned to assist and expedite cross-border vendors in meeting these requirements.

In light of misleading claims and information disseminated by influencers and live streamers lacking relevant expertise, the Malaysian Communications and Multimedia Commission may collaborate with the Royal Malaysia Police and the MOH to take action against those spreading misleading information under the Communications and Multimedia Act 1998. Moreover, the public should be educated to fact-check any doubtful information through the government-operated website (sebenarnya.my)^(19)^. Influencers and live streamers who intend to disseminate health-related claims or opinions on digital platforms should be legally required to obtain prior endorsement of such content from a registered nutritionist or dietitian. To ensure professional accountability, the practising registration number of the endorsing nutritionist or dietitian should be clearly displayed on screen during the livestream or recorded content. In the future, registered nutritionists and dietitians may be trained to serve as key opinion leaders in digital nutrition communication to publicise accurate health-related information.

### Recommendation 5: Behavioural nudging, interface redesign and technological interventions to promote healthier choices on digital platforms

Digital platform operators can work closely with nutritionists to redesign the interface of digital platforms to make healthier options more prominent. This can be achieved through a range of strategies, for instance, incorporating traffic light labels, the Healthier Choice Logo and nutrition taglines on product pages to guide consumers toward healthier choices. Healthier options should be prioritised by default, with nutrient-dense foods positioned at the top of selection lists to enhance their visibility, rather than relying solely on popularity-based algorithms. The online food delivery applications should also consider integrating digital dietary tracking tools (such as the MyNutriDiari 2) within the platform to enable users to plan their intakes based on daily nutrient requirements. In view of recent developments in artificial intelligence, digital platform operators should consider incorporating artificial intelligence-driven personalised dietary recommendations that align with individual nutritional goals.

## Conclusion

Although shaping a healthier digital food environment presents substantial challenges, it is achievable through the nutrition-conscious actions outlined above. Fostering such an environment requires collaboration among government ministries, platform operators, online food vendors and platform users.
